# Intercellular communication is a heritable dimension of human tissue architecture

**DOI:** 10.64898/2026.03.29.715138

**Published:** 2026-06-04

**Authors:** Chen Yang, Xianyang Zhang, Jun Chen

**Affiliations:** 1 Department of Statistics, Texas A&M University, College Station, Texas, 77843, USA.; 2 Division of Computational Biology, Department of Quantitative Health Sciences, Mayo Clinic, Rochester, Minnesota, 55905, USA.

**Keywords:** heritability partitioning, spatial transcriptomics, cell–cell communication, intercellular signaling genetics, ligand–receptor interactions

## Abstract

Methods that map genetic risk to cells identify disease-relevant tissues and cell types but cannot test whether genetic effects concentrate at molecular interfaces between cells. Here we introduce EdgeMap, which integrates spatial transcriptomics with GWAS summary statistics to partition trait heritability into cell-intrinsic and intercellular components and to resolve the intercellular signal into specific ligand–receptor channels. Across 17 traits and five human tissues, edge heritability is enriched in biologically coherent trait–tissue pairings (3.8-fold; *P* **= 4**.**4** *×* **10**^*−***6**^) and replicates across independent tissue sections, GWAS cohorts, and cell-segmented Visium HD. Per-pair decomposition identifies 67 trait-specific channels (FDR < **0**.**10**) organized into convergent pathway families—neurexin–neuroligin synaptic signaling in bipolar disorder, vascular adhesion in cardiovascular traits, and lipoprotein-clearance pathways in liver. Most edge genes are absent from standard gene-level prioritization, supporting intercellular communication as a complementary dimension of genetic architecture.

## 1 Introduction

Genome-wide association studies (GWAS) have identified thousands of loci for complex traits, but linking these associations to the cellular programs through which they act remains a central challenge[1]. A major advance has come from partitioning SNP heritability across biologically informed annotations. Stratified LD score regression (S-LDSC)[2], which regresses GWAS association statistics on annotation LD scores, localizes heritability to functional categories; cell-type-specific extensions map heritability to trait-relevant tissues and cell populations[3, 4]; scDRS[5] resolves signal at single-cell resolution; and gsMap[6] extends this logic to spatial domains within tissues. Together, these approaches have made genetic risk increasingly interpretable by revealing *where* within a tissue heritability concentrates and *which* cellular contexts are most implicated.

Yet tissues are not organized solely by cell identity; they are also shaped by communication between neighboring cells. Over 4,000 ligand–receptor (LR) pairs mediate these interactions[7–9], and spatial transcriptomics now makes them measurable *in situ*[10–12]. This biology is directly relevant to human genetics: GWAS loci are enriched for genes encoding interacting protein partners[13], suggesting that genetic risk has a relational component that extends beyond cell-autonomous gene expression.

Despite this relevance, heritability partitioning remains fundamentally cell-centric. S-LDSC, scDRS and gsMap—which assigns GWAS signal to individual genes via a genome-wide SNP–gene weight matrix based on LD and gene proximity, without aggregating information across spatial neighbors—treat each cell or spatial location as an independent unit whose own expression profile determines its contribution to heritability. These methods can identify trait-relevant tissues and cellular contexts, but they cannot test whether genetic risk is concentrated in the signaling pathways that connect neighboring cells. If a variant perturbs a ligand in one cell and its phenotypic consequence depends on a receptor in another, the relevant intercellular *edge* is absorbed into cell-intrinsic signal rather than resolved as a distinct mechanism.

**Fig. 1:**
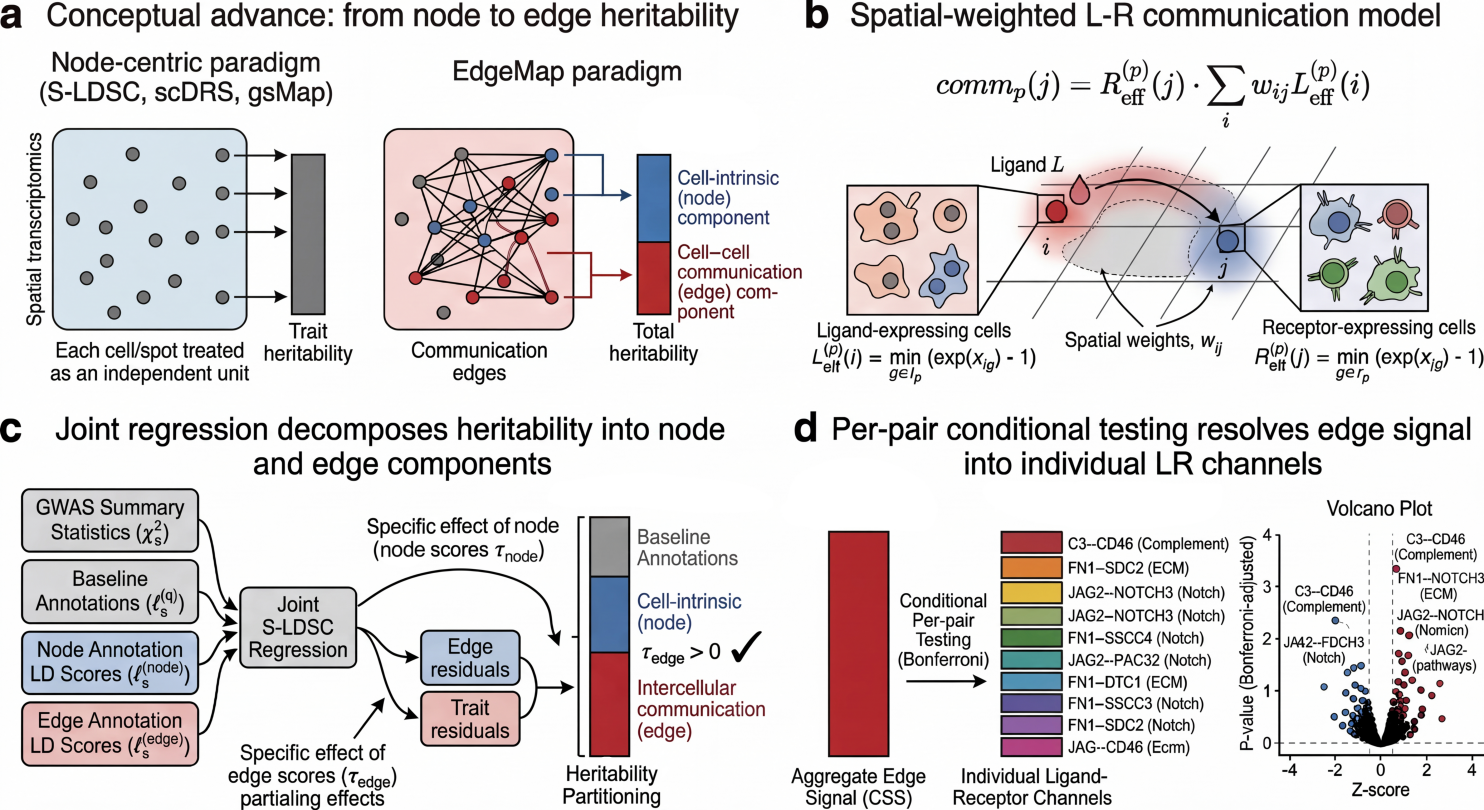
Overview of the EdgeMap framework. **a**, Conceptual advance: node-centric methods (S-LDSC, scDRS, gsMap) identify disease-relevant tissues and cell types but treat each cell independently; EdgeMap further decomposes heritability into cell-intrinsic (node) and cell–cell communication (edge) components, resolving which intercellular channels carry genetic risk. **b**, Spatial-weighted communication model for ligand–receptor pairs. **c**, Joint regression decomposes heritability into node and edge components via Frisch–Waugh–Lovell. **d**, Per-pair conditional testing resolves edge signal into individual LR channels.

Here we introduce EdgeMap, which explicitly decomposes trait heritability into **node** (cell-intrinsic) and **edge** (cell–cell communication) components (Fig. 1). From spatial transcriptomics data we derive two parallel gene-level annotations—one capturing cell-intrinsic expression, the other capturing spatially concentrated ligand–receptor signaling. Both are mapped to SNP-level LD scores and entered jointly into a regression framework, where the Frisch–Waugh–Lovell theorem[14, 15] guarantees that each coefficient captures unique variance after partialling out all other annotations. Per-pair conditional testing then resolves the aggregate edge signal into individual ligand–receptor channels—a level of pathway-specific resolution that node-centric frameworks do not currently offer. Applied across 17 complex traits and five human tissues, we show that this decomposition yields biologically distinct gene sets, is consistent across independent tissue sections, and identifies communication channels that partially overlap current drug targets while recovering known disease biology.

## 2 Results

### 2.1 The EdgeMap framework

EdgeMap takes spatial transcriptomics data and GWAS summary statistics as input (Fig. 1). For each tissue, we construct a spatial neighbor graph and compute per-cell communication intensity for each ligand–receptor (LR) pair in the LIANA Consensus resource[7] (4,624 curated interactions; Methods). Existing communication inference tools[16, 17] estimate interaction activity at the level of cell pairs or individual channels—a format suited for communication biology but not for genome-wide heritability annotation. EdgeMap bridges this gap by deriving two parallel gene-level scores: the **gene specificity score** (GSS, hereafter *node score*) quantifies how spatially concentrated a gene’s expression is; the **communication specificity score** (CSS, hereafter *edge score*) quantifies how spatially concentrated its LR communication is. Both are mapped to SNP-level annotation LD scores and entered jointly into the EdgeMap regression alongside baseline annotations. A significant positive edge coefficient $\tau_{\text{edge}}$ indicates that SNPs near communication-active genes carry excess heritability beyond what cell-intrinsic and baseline annotations explain. Per-pair conditional testing further decomposes the edge signal into individual LR channels, calibrated against an empirical null to account for the sparse-annotation regime (Methods). Because the LIANA Consensus resource includes some pairs whose partners lack documented extracellular localization, all biological interpretation of per-pair results uses extracellular-verified pairs only (tiers 1 and 2; Methods, §4.28); results using the full database are reported as a sensitivity analysis (Supplementary Table 6).

### 2.2 Simulations confirm calibration and node–edge separation

**Fig. 2:**
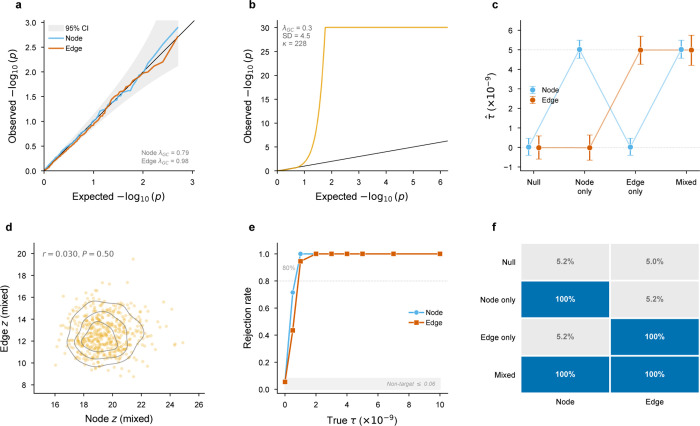
Simulation validation (heart annotation LD scores; brain and gut in Extended Data Table 3). **a**, Aggregate null QQ plot (n=500 replicates): node and edge *P*-values are well calibrated. **b**, Per-pair null QQ plot: sparse pair annotations generate a heavy-tailed null that departs strongly from N(0,1), motivating empirical calibration. **c**, Coefficient recovery: estimated $\hat{\tau }$ (median ± 95% interval) versus true τ across all scenarios; bias <0.6%. **d**, Node and edge *z-*scores are uncorrelated under the mixed architecture (r=0.030,P=0.50), confirming independent decomposition. **e**, Power curve: rejection rate as a function of true effect size τ ; the target component reaches 100% power by $\tau =2\times 10^{-9}$ while the non-target component maintains nominal error. **f**, Rejection-rate summary across null, node-only, edge-only, and mixed architectures, showing calibrated type-I error and complete power in the targeted non-null settings.

We validated EdgeMap using real annotation LD scores from spatial transcriptomics with synthetic GWAS $\chi^{2}$ statistics across four architectures (null, node-only, edge-only, mixed; 500 replicates each; Fig. 2). Simulations were conducted on three tissues (heart, brain, gut) to confirm that calibration generalizes across annotation structures (Extended Data Table 3); results below are from heart.

Under the null, type-I error was well calibrated: 5.2% for node and 5.0% for edge at *α* = 0.05 (*P*-values uniform by Kolmogorov–Smirnov test). Under node-only enrichment, the node component reached 100% power (mean *z* = 19.5) while the edge component maintained nominal error (5.2%). Under edge-only enrichment, the pattern reversed exactly: edge at 100% power (mean *z* = 13.6), node calibrated (5.2%). This clean separation—zero leakage between annotations—validates the joint regression as a decomposition of heritability into node and edge components. Null type-I error ranged from 4.4% to 6.2% across all three tissues (Extended Data Table 3), confirming that aggregate calibration is robust to differences in annotation structure. Per-pair annotations are much sparser (∼1,000 nonzero SNPs per pair), producing null *z*-scores that depart from *N*(0,1) (sd ≈ 4.4, excess kurtosis 50–5,000; Fig. 2b). We therefore calibrate per-pair significance via 50,000-replicate empirical null simulation per tissue (Methods, §4.8), yielding assumption-free empirical *P*-values. Per-pair *z*score rankings are robust to calibration method (mean Spearman *ρ* = 0.84 between |z| rank and empirical *P* rank; Extended Data Fig. ED4c). Importantly, because per-pair null *z*-distributions are shaped by annotation sparsity and are generally not centered at zero, empirical *P*-values measure enrichment relative to the pair-specific null regardless of the sign of the raw *z*-score: a pair with negative raw *z* can be significant if its null distribution is shifted even more negatively (Methods, §4.8).

### 2.3 Communication heritability landscape across traits and tissues

**Fig. 3:**
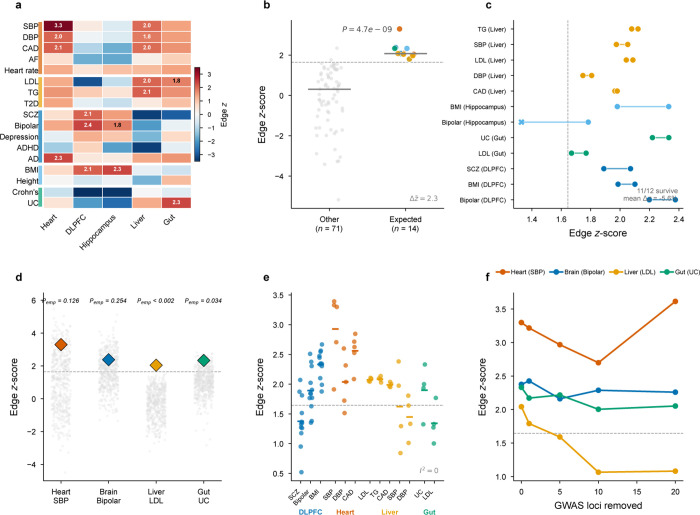
Global evidence for communication heritability. **a**, Heatmap of edge *z*-scores across 85 trait–tissue combinations. Stars mark significant combinations (*P* < 0.05, one-sided); colour intensity reflects *z*-score magnitude. **b**, Biological coherence: edge *z*-scores in *a priori* expected trait–tissue pairings versus all others (Mann–Whitney *P* = 4.7 × 10^−9^; ∆*z* = 2.29). **c**, Cell-type composition control: paired edge *z*-scores before versus after conditioning on spatial-domain gene-expression annotations. 11 of 12 significant combinations survive the additional control (mean ∆*z* = −5.6%). **d**, LR database specificity: real edge *z*-scores (diamonds) versus 500 expression-matched shuffled LR databases (dots). Liver (*P*_emp_ < 0.002) and gut (*P*_emp_ = 0.034) show significant LR specificity; heart and brain are marginal (see Discussion). **e**, Cross-section replication: edge *z*-scores across independent tissue sections (12 brain DLPFC sections from 3 donors, 5 heart sections from 5 donors, 4 liver sections from 4 donors, 4 gut sections from 4 donors). **f**, Locus ablation: edge *z*-scores after removing the top *K* GWAS loci (±500kb). Heart, brain, and gut maintain significance at all ablation levels; liver attenuates beyond 5 loci, consistent with concentrated lipoprotein-pathway architecture.

To test whether communication heritability is a systematic phenomenon rather than a property of individual trait–tissue pairs, we applied EdgeMap to 17 complex traits spanning cardiovascular, psychiatric, metabolic, and immune categories across five human tissues: heart (Visium, 4,247 spots, 479 active LR pairs), brain DLPFC (section 151507 from Maynard et al. 2021[18]; 4,226 spots, 346 active pairs), hippocampus (section V10B01–085 A1 from Thompson et al. 2025[19]; 4,992 spots, 691 active pairs), liver (section JBO001 from Guilliams et al. 2022[20]; 1,646 spots, 779 active pairs), and intestine (section B4; 2,575 spots, 231 active pairs; Fig. 3; Supplementary Table 1).

Of 85 trait–tissue combinations, 16 showed significant edge heritability (*P* < 0.05, one-sided)—3.8-fold over the ∼4.25 expected by chance (binomial *P* = 4.4 × 10^−6^). Under two-sided testing, 13 of these 16 retained significance, while 9 biologically unexpected pairings showed significant *depletion* of communication heritability (negative *z*; e.g., Crohn’s disease in brain, *z* = −5.17; schizophrenia in liver, *z* = −3.27; Supplementary Table 7)—a bidirectional pattern consistent with biological specificity rather than annotation noise. Trait-label permutation (*P* < 10^−4^), which preserves the cross-trait dependence structure within each tissue, and cross-section random-effects meta-analysis (Extended Data Table 1; §2.5) supported the same conclusion. Individual trait–tissue *z*-scores are moderate (*z* ≈ 2–3), as expected when aggregate annotations carry limited per-SNP variance in the S-LDSC regime[2]; only one combination survives Benjamini–Hochberg correction at FDR < 0.05 (*q*_min_ = 0.041). The primary evidence for communication heritability therefore lies in the landscape-level concentration of signal across the full trait–tissue panel rather than in any single aggregate test.

These 16 aggregate-significant combinations concentrated in biologically expected trait–tissue pairings defined *a priori* (14 of 16; cardiovascular traits in heart and liver, psychiatric traits in brain, metabolic traits in liver, IBD in gut; Methods): Stouffer combination across the 14 expected pairings yielded *z* = 8.09 (*P* = 2.9×10^−16^; Mann– Whitney *P* = 4.7×10^−9^; Fig. 3b), and the signal remained significant after removing any single tissue (leave-one-tissue-out *P* < 6 × 10^−4^ for all five). Trait-label permutation confirmed this concentration: shuffling labels 10,000 times, the null expectation was 3.1 significant results in expected pairings versus the 14 observed (*P* < 10^−4^; ∆*z* = 2.29, never matched under permutation). The decomposition also revealed trait-dependent architecture: atrial fibrillation, which includes major risk loci in cardiomyocyte ion-channel genes such as SCN5A and KCNQ1, showed no edge signal in any tissue (*z* = 0.58 in heart); T2D, which includes strong cell-intrinsic regulatory loci such as TCF7L2 and HNF1A, was similarly node-dominated (*z* = 0.34 in liver). This trait-dependent pattern—edge enrichment for communication-intensive traits but not for ion-channel or transcription-factor-driven traits—indicates that EdgeMap captures a specific component of genetic architecture rather than indiscriminate annotation bias. Two further controls corroborate these findings. Replacing the LIANA Consensus database with 500 expression-matched shuffled databases confirmed that the edge signal requires known LR biology in liver and gut, while heart and brain showed marginal specificity—reflecting concentrated signaling repertoires (Fig. 3d; Discussion). Greedy locus ablation confirmed polygenic distribution in heart, brain, and gut; liver attenuated beyond five loci, consistent with concentrated lipoprotein-pathway architecture (Fig. 3f).

### 2.4 Node and edge components capture distinct genetic signals

A natural concern is whether the edge component merely re-identifies the same genes that drive node heritability. We tested this directly by comparing the genes in per-pair-significant edge pairs with the top-ranked genes by node specificity score (top 100 per tissue; Methods). Across the trait–tissue combinations that yielded significant LR pairs in per-pair testing, the mean Jaccard overlap was 0.002 (median 0), with only 2.1% of edge-pair genes appearing among the top node genes (Extended Data Fig. ED3a). Edge-pair genes showed only weak node-score enrichment (mean rank-biserial correlation = 0.09; Mann–Whitney *P* > 0.05 in 10 of 12 combinations), and genome-wide node and edge scores were essentially uncorrelated (mean Spearman *ρ* = 0.02). At the significance level, all 16 edge-significant trait–tissue combinations are also node-significant, consistent with the expectation that communication heritability arises within trait-relevant tissues. Together with the simulation results (§2.2), these analyses indicate that EdgeMap’s node and edge components capture distinct aspects of heritability: both contribute, but they operate through largely non-overlapping gene sets and thus capture complementary dimensions of genetic architecture (Extended Data Fig. ED3a).

This complementarity extends beyond EdgeMap’s own node component. Comparing edge genes against gene-level association testing analogous to MAGMA[21] (minimum SNP *P* within ±10kb, Bonferroni threshold *P* < 2.5×10^−6^) and Open Targets Locus-to-Gene fine-mapping[22] (L2G score ≥ 0.5, matched per trait; Methods), 46 of 72 edge genes (64%) were identified by neither method (Extended Data Fig. ED5; Supplementary Table 8). These include genes with clear disease-relevant biology that emerges only in the communication context: CNTNAP1, a paranodal junction protein whose RTN4–CNTNAP1 pair is the sole Bonferroni-significant edge channel for bipolar disorder in brain; GPC3, a glypican co-receptor whose loss-of-function causes an over-growth syndrome with congenital cardiac defects[23], identified through GPC3–FLT1 and GPC3–LRP1 pairs for blood pressure in heart; and F9 (coagulation factor IX), whose hepatocyte-secreted ligand signals to stellate-cell LRP1 for LDL heritability in liver (§2.7). None of these genes reaches genome-wide significance individually; their heritability is visible only through the LR pair annotation. The pattern is consistent across tissues and reflects a fundamental difference in selection criteria: edge genes are prioritized by communication-based annotation enrichment rather than by proximity to GWAS lead variants.

We also tested whether the edge signal reflects spatial variation in cell-type composition rather than communication *per se*. Augmenting the S-LDSC regression with spatial domain annotations that capture local cellular composition (Leiden-clustered neighborhoods; Methods, §4.25) had minimal impact: 11 of 12 tested edge-significant combinations (91.7%) retained significance, with a mean *z*-score change of only −5.6% (Extended Data Table 2). Communication heritability is thus not reducible to composition gradients.

### 2.5 Communication heritability replicates across tissue sections and independent GWAS

To test whether edge signals reflect stable tissue biology rather than section-specific artifacts, we ran EdgeMap independently on multiple sections per tissue (12 brain DLPFC from 3 donors[18], 5 heart from 5 donors[24], 4 liver from 4 donors[20], 4 gut from 4 donors; Fig. 3e). All significant trait–tissue combinations replicated with $I^{2}=0$ (random-effects meta-analysis; Extended Data Table 1), with meta-analytic *z*-scores ranging from 3.80 (gut UC) to 8.08 (brain BMI; all meta *P* < 10^−4^). Section-level edge *z*-scores were consistently positive across independent tissue sections (Extended Data Fig. ED3c). Communication intensity profiles were concordant within brain, liver, and heart (*ρ* = 0.53–0.87); gut showed negligible profile concordance (*ρ* ≈ −0.01) despite consistently elevated aggregate heritability, consistent with heterogeneous mucosal architecture. Per-pair *z*-score vectors were near-perfectly concordant across sections (*ρ* = 0.983–0.992), with trait-specific rather than annotation-driven structure (within-trait *ρ* = 0.99 versus cross-trait *ρ* = 0.34).

#### Independent GWAS replication.

Replacing the primary LDL GWAS (UK Biobank) with a fully independent dataset (GLGC 2013[25]; *N* = 173,082; no sample overlap), the liver edge signal replicated (*z* = 2.24, *P* = 0.013), with per-pair *z*-scores concordant between the two GWAS (Spearman *ρ* = 0.53, *P* = 1.7 × 10^−50^; Fig. 5d) and PCSK9–SORT1 among the top channels in both. An analogous test for heart using FinnGen Release 10 CAD[26] (*N*_eff_ = 166,436; no sample overlap) also replicated (*z* = 1.94, *P* = 0.026).

#### Cross-platform validation.

EdgeMap applied to cell-segmented Visium HD liver data[27] (8*µ*m resolution; independent donors and laboratory) confirmed the edge signal (*z* = 1.93, *P* = 0.027; Fig. 5e). At native single-cell resolution, all five top-ranked liver–LDL pairs showed distinct primary sender and receiver cell types—hepatocyte-secreted F9 signaling to stellate-cell LRP1, hepatocyte C3 to cholangiocyte C3AR1 (Fig. 5c)—consistent with intercellular rather than autocrine communication.

### 2.6 Cardiovascular communication pathways carry blood pressure heritability

We next examined per-pair results in individual tissues, using empirical null calibration (§2.2, §4.8). We distinguish *discoveries* (Bonferroni-significant under the empirical null) from *candidates* (FDR < 0.10), the latter providing hypothesis-generating context. Heart, which showed the strongest aggregate edge signal, tests whether top-ranked pairs converge on coherent pathway biology or scatter randomly across the LR database.

For systolic blood pressure (SBP, *N* = 340,159), the node annotation was highly significant (*z* = 3.21, *P* = 6.6 × 10^−4^), consistent with prior findings[6]. The edge annotation was independently significant (*z* = 3.30, *P* = 4.9 × 10^−4^; *τ*_edge_ = 8.58 × 10^−9^, SE = 2.60 × 10^−9^).

**Fig. 4:**
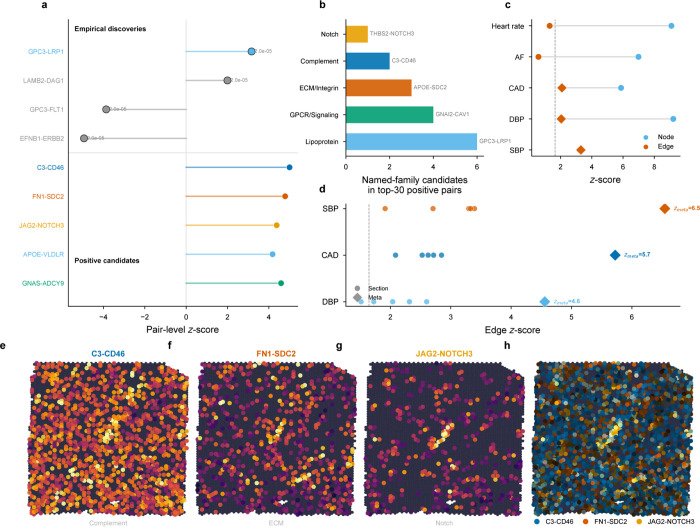
Cardiovascular communication pathways carry blood pressure heritability. **a**, Ranked pair-level summary for heart–SBP. The upper band shows the four empirical Bonferroni discoveries; the lower band shows biologically interpretable positive candidates highlighted for pathway-level follow-up. **b**, Pathway convergence: named-family candidates among the top positive heart–SBP pairs collapse onto a small number of recurrent pathway families rather than scattering across the LR database. **c**, Multi-trait comparison: SBP, DBP, and CAD show significant edge signal; AF and heart rate serve as negative controls. **d**, Cross-section replication across 5 independent heart sections from 5 donors for SBP, CAD, and DBP. **e–h**, Spatial communication maps for three top SBP pairs from distinct pathway families: complement (C3–CD46), ECM (FN1–SDC2), and Notch (JAG2–NOTCH3). Rightmost panel: RGB composite overlay demonstrating spatial separation of pathway-specific communication niches.

Per-pair decomposition of the SBP edge signal (370 active pairs, 367 empirically testable; empirical null calibration, Methods) identified four Bonferroni-significant channels (*P*_emp,Bonf_ < 0.05): EFNB1–ERBB2, GPC3–FLT1, GPC3–LRP1, and LAMB2–DAG1 (Fig. 4a; Supplementary Table 2). These channels converge on cell-surface growth factor and adhesion receptor signaling. Beyond these four discoveries, we highlight top-ranked extracellular-verified pairs that span a broader range of pathway families than the Bonferroni set alone: (i) complement signaling (C3–CD46, *z* = 5.00), (ii) fibronectin–ECM signaling (multiple FN1-receptor pairs), (iii) Notch vascular signaling (JAG2/PSEN1/THBS2–NOTCH3; *NOTCH3* mutations cause CADASIL[28, 29]), (iv) GPCR signaling (GNAS/GNAI2–ADCY9, the Gs/Gi–cAMP axis), and (v) lipoprotein receptor signaling (APOE–VLDLR).

Diastolic blood pressure (*z* = 2.04; 2 empirical Bonferroni pairs) and coronary artery disease (*z* = 2.09; 3 empirical Bonferroni pairs) were independently significant in heart, consistent with shared cardiovascular communication biology. Alzheimer’s disease also reached significance in heart (*z* = 2.34, *P* = 0.010), with the top pair F8–LRP1 (*P*_emp,Bonf_ = 0.007) converging on LRP1-mediated clearance—a cross-tissue signal discussed further in §3.

The edge signal was robust across spatial neighborhood sizes *k* ∈ {4, 6, 10, 15, 20} and communication specificity thresholds *P*_%_ ∈ {85, 90, 95, 99} (significant in all 20 tested parameter combinations; Extended Data Fig. ED3b).

### 2.7 Liver recovers PCSK9 biology and reveals metabolic communication

Liver offers a different test: because the PCSK9–SORT1 axis is among the best-characterized genetic mechanisms for any complex trait, liver–LDL provides a benchmark for whether EdgeMap recovers *established biology*. LDL cholesterol showed significant edge heritability (*z* = 2.04, *P* = 0.021). Per-pair decomposition with empirical null calibration identified 10 Bonferroni-significant pairs out of 664 tested (668 active) (Supplementary Table 4). Among these, ICAM1–IL2RG links intercellular adhesion to cytokine receptor signaling. F9–LRP1 and PLG–IGF2R (both *P*_emp,Bonf_ = 0.013) implicate the hepatic clearance receptor axis, while RSPO3–RNF43 (*P*_emp,Bonf_ = 0.013) acts through the R-spondin–Wnt module that establishes the portal–central metabolic zonation of the liver itself[30]—a mechanistic loop in which the same intercellular axis that patterns hepatic lipid metabolism also carries LDL heritability, and C3–C3AR1 (*P*_emp,Bonf_ = 0.013) implicates complement-mediated communication. Beyond these Bonferroni discoveries, the broader per-pair landscape converges on hepatic lipoprotein clearance: PCSK9–SORT1 ranked 5th by raw *z*-score (*z* = 5.71, *P*_emp_ = 0.044; not individually Bonferroni-significant) and recapitulates a well-characterized LDL regulatory axis targeted by evolocumab and alirocumab—SORT1 facilitates intracellular trafficking and secretion of PCSK9[31], which degrades LDL receptors on neighboring hepatocytes. The SORT1 locus (1p13.3) is one of the strongest common-variant signals for LDL cholesterol[32].

Triglycerides were independently significant (*z* = 2.08, *P* = 0.019; 3 empirical Bonferroni pairs), and blood pressure traits also reached significance (SBP *z* = 1.98, 13 empirical Bonferroni pairs; DBP *z* = 1.81, 2 pairs), reflecting the cardiometabolic continuum. CAD (*z* = 1.97, *P* = 0.025; 1 empirical Bonferroni pair) yielded F8–LRP1 as its sole empirical discovery, implicating the LRP1 clearance axis.

Top-ranked LR pairs showed a 1.84-fold enrichment for spatial gradients along the portal–central axis (permutation *P* < 0.001; Extended Data Fig. ED2f), consistent with the known zonation of hepatic lipid metabolism[33].

**Fig. 5:**
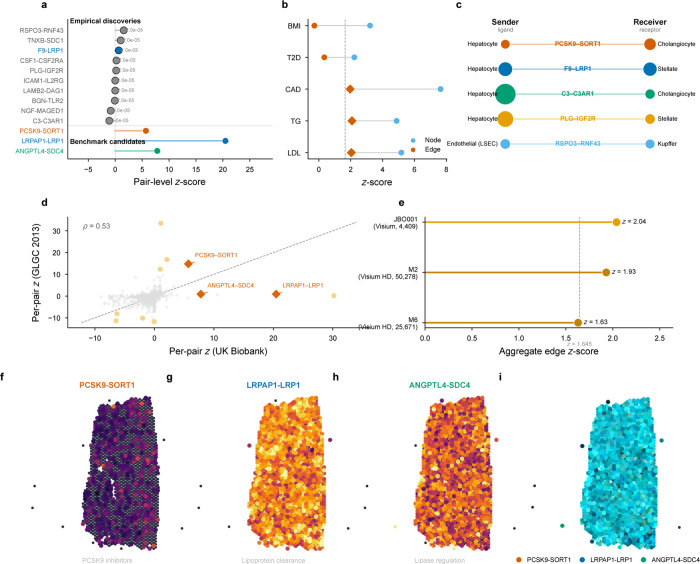
Liver recovers PCSK9 biology and reveals intercellular lipid communication. **a**, Two-band pair-level summary for liver–LDL. The upper band shows empirical Bonferroni discoveries, whereas the lower band highlights mechanistically relevant candidates including PCSK9–SORT1, LRPAP1–LRP1, and ANGPTL4–SDC4. **b**, Multi-trait comparison in liver: LDL, TG, CAD show significant edge signal; T2D and BMI show node-dominant, edge-nonsignificant patterns. **c**, Sender–receiver resolution in Visium HD M6: top liver LDL pairs show distinct primary sender and receiver cell types, supporting intercellular rather than autocrine signaling. **d**, Independent GWAS replication: per-pair *z*-scores in liver for UK Biobank LDL (x) versus GLGC 2013 LDL (y; no sample overlap). PCSK9–SORT1 is among the top-ranked channels in both GWAS independently. **e**, Cross-platform validation: aggregate edge *z*-scores for LDL in liver using standard Visium (∼55*µ*m) and cell-segmented Visium HD (∼8*µ*m) from independent donors and laboratories. **f–i**, Spatial communication maps for the three top LDL pairs: **f**, PCSK9–SORT1 (PCSK9 inhibitors), **g**, LRPAP1–LRP1 (tier 3; lipoprotein clearance locus), **h**, ANGPTL4–SDC4 (lipid metabolism), and **i**, RGB composite overlay.

As expected from their cell-intrinsic genetic architectures (§2.3), T2D and BMI showed no edge signal in liver (Fig. 5b).

### 2.8 Trait-specific communication signatures in brain

**Fig. 6:**
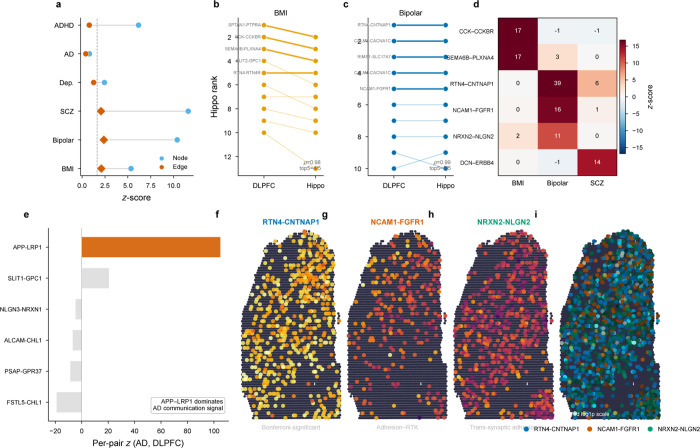
Trait-specific communication signatures in brain. **a**, Node and edge *z*-scores across traits in DLPFC: BMI, bipolar, and SCZ show significant edge signal; depression and AD do not. **b–c**, Cross-region rank concordance between DLPFC and hippocampus for BMI (**b**) and bipolar disorder (**c**): top-ranked pairs are preserved across brain regions. **d**, Trait specificity heatmap: top pairs for each trait show near-zero or negative *z*-scores for other traits, demonstrating trait-specific communication architecture. **e**, Alzheimer’s disease focused view: APP–LRP1 dominates the pair-level brain AD signal, consistent with a concentrated amyloid-clearance channel rather than distributed edge enrichment. **f–i**, Spatial communication maps for three top extracellular-verified bipolar pairs: RTN4–CNTNAP1 (the sole Bonferroni-significant pair), NCAM1–FGFR1 (adhesion–RTK), and NRXN2–NLGN2 (trans-synaptic adhesion). Rightmost panel: RGB composite overlay showing spatial separation of the three signaling programmes across cortical territory.

Heart and liver each yielded a single dominant trait–tissue story; brain tests whether the edge signal is *trait-specific* within a single tissue. If communication heritability were a tissue-level confound, every trait would engage the same LR pairs. The brain DLPFC provided a direct test: three traits showed significant edge heritability in the *same tissue* using the *same* LR database, yet each yielded a distinct set of top channels (Fig. 6).

#### BMI.

BMI showed significant edge heritability (*z* = 2.10, *P* = 0.018); top-ranked pairs included CCK–CCKBR (*z* = 17.35), encoding the cholecystokinin appetite– satiety system[34], and SEMA6B–PLXNA4 (*z* = 16.53), implicating axon guidance.

#### Bipolar disorder: adhesion-mediated synaptic signaling.

Bipolar disorder showed the strongest edge signal among psychiatric traits (*z* = 2.38, *P* = 8.8×10^−3^). Per-pair decomposition with empirical null calibration identified one Bonferroni-significant pair (RTN4–CNTNAP1, *z* = 39.19, *P*_emp_ = 6.0 × 10^−5^, *P*_emp,Bonf_ = 0.013) and 19 pairs at FDR < 0.10 (Supplementary Table 3). RTN4 encodes Nogo-A, an oligodendroglial protein that directly binds axonal Caspr (CNTNAP1) in *trans* at CNS paranodes[35], forming the molecular junction that segregates K^+^ channels and maintains saltatory conduction. This finding converges with robust evidence for oligodendrocyte and myelin gene downregulation in bipolar cortex[36] and with the genome-wide significant association of *ANK3*, whose protein ankyrin-G is required for paranodal junction assembly on the glial side. After subcellular localization quality control (Methods, §4.28), FDR-level candidates organized into three convergent pathway families. (i) *Adhesion–receptor tyrosine kinase signaling.* NCAM1–FGFR1 (*z* = 15.88, *P*_emp,FDR_ = 0.08) and L1CAM–CD9 (FDR = 0.07) link neural cell adhesion molecules to membrane receptors at the cell surface. NCAM1 is a well-characterized FGFR1 ligand whose binding activates neurite outgrowth signaling[37]. L1CAM also interacts with ERBB2 via its Ig-like domains[38], coupling cell adhesion to the neuregulin–ErbB signaling axis implicated in synaptic plasticity[39]; although L1CAM–ERBB2 ranks highly (*z* = 28.21), its sparse annotation precludes reliable individual significance estimation (§4.8). (ii) *Trans-synaptic adhesion.* NRXN2–NLGN2 (*z* = 10.95, FDR = 0.07) implicates the neurexin–neuroligin family, whose members mediate excitatory and inhibitory synapse organization and have been implicated across psychiatric conditions including autism spectrum disorder and schizophrenia[40]. (iii) *Secreted signaling.* EFNB3–EPHA4 (*z* = 6.59, FDR = 0.07) and MDK–SORL1 (FDR = 0.07) contribute ephrin contact guidance and midkine– SorLA signaling, respectively. Pairs involving calmodulin (CALM3–CACNA1C, *z* = 34.03, FDR = 0.07) reflect the strong GWAS signal at the *CACNA1C* locus; calmodulin binds CACNA1C at the cytoplasmic IQ domain[41] rather than across the cell surface, and these pairs are flagged as localization-unverified (§4.28).

#### Schizophrenia.

Schizophrenia showed significant edge heritability (*z* = 2.07, *P* = 0.019) with no individual Bonferroni pair. Top-ranked channels included DCN–ERBB4 (*z* = 13.95), implicating the neuregulin–ErbB signaling pathway[39, 42], and NLGN2–NRXN1 (*z* = 4.83), reinforcing the neurexin–neuroligin axis identified for bipolar disorder.

Across all three traits, the brain channels converge on contact-dependent adhesion molecules—neurexin–neuroligin trans-synaptic complexes, neural cell adhesion molecules (NCAM1, L1CAM) activating receptor tyrosine kinases, and ephrin contact guidance—indicating that synaptic and peri-synaptic signaling constitutes the principal communication channel family for psychiatric traits in cortex. This convergence is independently supported by rare-variant genetics: *NRXN1* deletions confer schizophrenia risk[43], and *NLGN3*/*NLGN4* mutations were among the first synaptic genes linked to autism-spectrum disorders[44].

The trait specificity of brain edge signals also extended across brain regions: in hippocampus, BMI replicated (*z* = 2.33, *P* = 9.9 × 10^−3^) with CCK–CCKBR again among the top channels (*z* = 25.89). Depression (*z* = 1.27, *P* = 0.10) and Alzheimer’s disease (*z* = 0.41) did not reach aggregate significance. For AD, however, per-pair analysis identified APP–LRP1 as Bonferroni-significant (*z* = 104.87, *P*_emp_ = 2.0 × 10^−5^), suggesting that the communication signal is concentrated in a single amyloid-clearance channel rather than distributed across many pairs—a pattern the aggregate annotation, which tests enrichment across all 217 channels simultaneously, lacks power to detect. The weaker common-variant signal (mean *χ*^2^ = 1.26 vs. 2.12 for SCZ) further limits aggregate sensitivity.

### 2.9 Ulcerative colitis engages mucosal adhesion channels

**Extended Data Fig. ED1:**
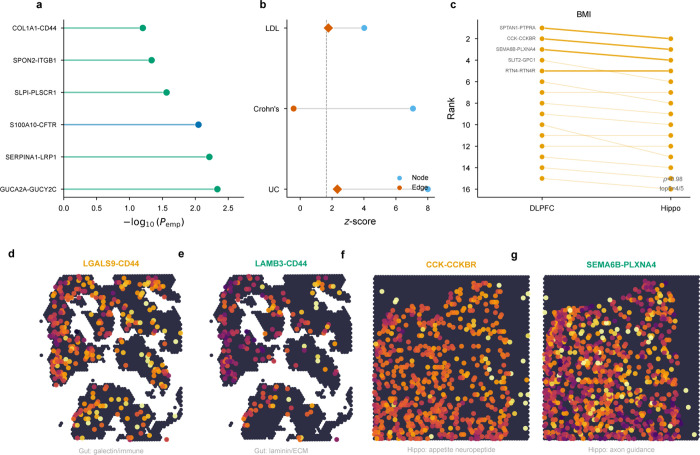
Gut and hippocampus secondary exemplars. **a**, Ranked empirical-*P* summary for gut × UC. The top candidate pairs remain below the empirical Bonferroni threshold, consistent with the smaller pair count in gut. **b**, Multi-trait comparison in gut: UC and LDL show significant edge signal; Crohn’s disease does not. **c**, Per-pair *z*-score comparison between DLPFC and hippocampus for BMI (r=0.64), confirming cross-region concordance of top communication channels. **d–e**, Representative gut maps for LGALS9–CD44 and LAMB3–CD44, highlighting epithelial–immune and laminin/ECM communication niches. **f–g**, Representative hippocampus maps for CCK–CCKBR and SEMA6B–PLXNA4, illustrating appetite-neuropeptide and axon-guidance signalling programmes.

Ulcerative colitis showed significant edge heritability in gut (*z* = 2.33, *P* = 9.9 × 10^−3^). No individual pair reached empirical Bonferroni significance out of 130 tested (142 active) (Supplementary Table 5), consistent with the smaller pair count. Top-ranked extracellular-verified pairs converge on CD44-mediated epithelial–immune adhesion: LGALS9–CD44 (*z* = 3.70) and LAMB3–CD44 (*z* = 3.44), consistent with CD44’s established role in mucosal leukocyte rolling and lymphocyte homing[45] (Extended Data Fig. ED1). That the leading edge pairs encode cell-surface adhesion molecules parallels the biology engaged by vedolizumab, which blocks integrin-mediated gut homing—a distinct molecular target acting on the same principle of immune-cell trafficking to the intestinal mucosa. Crohn’s disease did not reach significance (*z* = −0.41), as expected for transmural pathology poorly captured by mucosal Visium sections.

### 2.10 Biological content of edge genes

If edge genes represent a distinct dimension of genetic architecture, they should partially overlap with—but not recapitulate—existing gene-level annotations. We tested this prediction using two external reference annotations (drug target status, Mendelian disease genes) and a competitive gene-set test (Extended Data Fig. ED5c–e). Under empirical null calibration (§4.8), 67 LR pairs reached FDR < 0.10 across all tissue–trait combinations, yielding 72 unique edge genes (64 from tier 1 and tier 2 extracellular-verified pairs; Methods).

#### Drug target overlap.

Of 64 tier 1+2 edge genes, 34 (53.1%) are approved drug targets (DGIdb[46]; OR = 1.46, *P* = 0.086 versus the LR background; Extended Data Fig. ED5c), indicating modest, trait-dependent overlap with current pharmacology rather than an independent validation benchmark. Individual examples provide contextual anchors for prioritization: FLT1 (heart SBP) is the VEGFR1 axis underlying anti-angiogenic hypertension[47]; ERBB2 (heart SBP) mediates trastuzumab cardiotoxicity via endothelial NRG-1/ErbB2 signalling[48]; and LRP1 (brain AD; liver LDL) mediates both amyloid-*β* efflux and hepatic lipoprotein clearance.

#### Mendelian convergence.

One of 64 edge genes matched an OMIM Mendelian gene for the corresponding trait (APP for Alzheimer’s disease; OR = 0.41, *P* = 0.91). This is expected: Mendelian mutations typically disrupt cell-intrinsic protein function, whereas edge genes mark intercellular communication interfaces—a complementary biology. That APP—the causal gene for familial Alzheimer’s disease—nonetheless emerges as the sole Bonferroni-significant brain × AD pair (*P*_emp_ = 2.0 × 10^−5^) illustrates that per-pair decomposition can recover known biology when the communication signal concentrates in a single channel.

#### Competitive gene-set test.

Aggregating GWAS *P*-values to gene-level statistics (minimum *P* within ±10kb, analogous to MAGMA[21]), edge genes carried stronger associations than the LR background in 7 of 10 trait–tissue combinations (Wilcoxon rank-sum *P* < 0.05), with the strongest signals in liver × SBP (*P* = 2.2 × 10^−4^) and liver × LDL (*P* = 1.3 × 10^−3^), confirming that edge genes carry above-background GWAS signal within the LR database (this test shares GWAS input with EdgeMap and thus measures internal consistency; Methods).

**Extended Data Fig. ED2:**
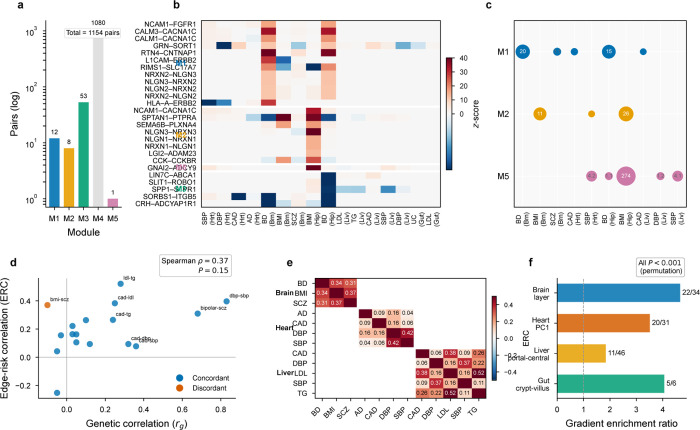
Edge module architecture and spatial organization. **a**, Module-size distribution across all 1,154 analysed pairs, showing that the edge landscape is dominated by one large background module and a smaller set of interpretable modules. **b**, Representative per-pair *z*-score heatmap across traits, ordered by module assignment. **c**, Module–trait loading heatmap: a synaptic module (M1, including NRXN–NLGN and NCAM1–FGFR) is selectively loaded on bipolar disorder; a neuropeptide module (M5, including CCK–CCKBR) is selectively loaded on BMI. **d**, Edge relevance correlation (ERC) vs. genome-wide genetic correlation $\left(r_{g}\right)$ ; Spearman *ρ* = 0.37. **e**, Within-tissue ERC heatmap showing pairwise trait correlations of per-pair edge *z*-scores (brain, heart, liver); LDL–TG in liver show the strongest cross-trait sharing (ERC = 0.52). **f**, Spatial gradient enrichment: fraction of top-ranked pairs (FDR < 0.10) exhibiting a significant spatial gradient along the tissue axis (cortical layer in brain, PC1 of spatial coordinates in heart, portal–central in liver, crypt–villus in gut).

Despite the statistical cost of testing sparse per-pair annotations (§4.8), pair-level profiles are highly reproducible across tissue sections, GWAS cohorts, and calibration methods (Extended Data Fig. ED4), indicating a stable biological signal. Clustering per-pair *z*-score profiles across traits organized these channels into five modules (Extended Data Fig. ED2): a synaptic module (NRXN–NLGN, NCAM1–FGFR) selective for bipolar disorder, a neuropeptide module (CCK–CCKBR) for BMI, and ECM/integrin and complement modules shared across cardiovascular traits—indicating that communication heritability is structured by pathway architecture.

## 3 Discussion

Across the settings examined here, complex-trait heritability is organized not only within cells but also across the molecular interfaces that connect neighboring cells. EdgeMap makes this intercellular component measurable, separable from cell-intrinsic effects, and resolvable into specific ligand–receptor channels.

Existing heritability methods—S-LDSC[2], scDRS[5], gsMap[6]—identify disease-relevant tissues and cell types but treat each cell as an independent scoring unit. EdgeMap adds a distinct layer of resolution. In heart, for example, node genes (NPPA, MYH7) are cardiomyocyte markers, whereas edge genes (EFNB1, GPC3, LAMB2, LRP1) encode intercellular signaling molecules that rank no higher in the node score distribution (§2.4).

This gap is not easily closed by applying existing communication tools directly to GWAS data. CellChat[16] and CellPhoneDB[17] produce cell-pair or interaction-level activity scores—outputs designed to characterize *which* cells communicate, whereas S-LDSC ultimately requires a genome-wide SNP-linked annotation. Converting interaction scores to gene-level annotations is therefore not a bookkeeping step: the aggregation rule determines which aspect of communication is encoded at the SNP level. EdgeMap’s CSS uses the maximum pair score across all LR pairs in which a gene participates, so each gene is annotated by its strongest communication channel before mapping to SNP-level LD scores for joint node–edge regression. The Frisch–Waugh–Lovell theorem then clarifies the interpretation of the edge coefficient after conditioning on node and baseline annotations. This same pair-to-gene construction also supports per-pair conditional decomposition within the heritability-partitioning framework, a use that existing interaction-level CCC scores do not directly provide.

A recurring observation across tissues is that communication heritability concentrates on pathway families: multiple independent receptor channels converge on fibronectin and Notch in heart, lipoprotein receptors in liver, and neurexin–neuroligin and adhesion–growth-factor-receptor cascades in brain. This convergence extends across tissues: LRP1, a pleiotropic clearance receptor, emerges as an edge gene paired with the tissue-appropriate ligand in each context—APP in brain×AD, F9 in liver×LDL, F8 in heart×CAD—consistent with its established tissue-specific clearance functions at the blood–brain barrier, in hepatocytes, and in the vasculature. The modular and cross-tissue organization of edge signals suggests that genetic risk at cell–cell interfaces is organized into discrete signaling modules rather than scattered across individual genes (Extended Data Fig. ED2), with biologically related traits sharing correlated edge profiles within tissues (Extended Data Fig. ED2c,e).

Two observations carry broader implications. First, the convergence of neural adhesion molecules (L1CAM, NCAM1) on receptor tyrosine kinases (ERBB2, FGFR1) in bipolar disorder (§2.8) illustrates that genetic risk at growth factor receptor loci may be enriched in genes encoding adhesion-dependent intercellular signaling interfaces rather than reflecting cell-autonomous receptor expression alone. Node-centric methods identify these genes individually; EdgeMap reveals that their heritability concentrates specifically in the spatial communication pattern between adhesion ligands and their receptors—a distinction not resolved by single-cell scoring. Second, edge signals are enriched for spatial gradients along tissue axes (§2.7, §2.8; Extended Data Fig. ED2f), implying that communication heritability respects tissue architecture rather than arising from spatially uniform annotation bias.

These properties—orthogonality, trait specificity, spatial organization—suggest that communication heritability can refine GWAS interpretation from “which tissues are relevant” to “which intercellular interfaces carry risk in those tissues.” Current GWAS-to-drug pipelines prioritize cell-intrinsic targets, yet the majority of approved drug targets are cell-surface or secreted proteins[49]. Drug-target catalogs are also historically shaped by tractability and therapeutic attention, so any overlap with edge genes should be read as contextual prioritization rather than validation. That 64% of edge genes are absent from standard gene-level prioritization (§2.4; Extended Data Fig. ED5) suggests that communication interfaces constitute an underexplored source of candidate targets with human genetic support.

The controls described in §2.4–2.3 (cell-type composition, gene-level cis-LD burden and expression, LR specificity, locus ablation; Fig. 3c,d,f) indicate that the edge signal is not reducible to a single confounding mechanism. The moderate cross-landscape correlation of node and edge *z*-scores (*ρ* = 0.61) is driven by between-tissue variance; within tissues it drops to *ρ* = 0.31 (*P* = 0.23 in heart), consistent with the expectation that communication heritability is engaged preferentially in trait-relevant tissues.

Several limitations should be noted. First, individual trait–tissue *z*-scores are moderate (*z* ≈ 2–3), as expected when aggregate annotations carry limited per-SNP variance in the S-LDSC framework[2]; the signal is confirmed at the landscape level (binomial *P* = 4.4 × 10^−6^; 13 of 16 retained under two-sided testing), by permutation, by coordinate noise injection showing that edge scores are invariant (*ρ* ≥ 0.97) even when node scores are destroyed (Extended Data Fig. ED7b), by cross-section meta-analysis (*P* < 10^−4^; Extended Data Table 1), by independent-GWAS replication (GLGC 2013 LDL, *P* = 0.013; FinnGen CAD, *P* = 0.026; §2.5), by cross-platform validation with cell-segmented Visium HD (*P* = 0.027; §2.5), by LR-database specificity testing (*P*_emp_ < 0.05 in two of four tissues), and by locus ablation confirming polygenic distribution in three of four tissues. Second, per-pair annotations are sparse, so block-jackknife standard errors lose *N*(0,1) validity (§4.8); we calibrate per-pair significance via 50,000-replicate empirical null simulation, and per-pair rankings are stable across calibration methods, tissue sections, and GWAS cohorts (Extended Data Fig. ED4), and conditional-null simulations with observed ${\hat{\tau }}_{\text{node}}$ confirmed that the global null provides adequate calibration (Extended Data Fig. ED4d,e; §4.8). Third, as noted in §2.1, the LIANA Consensus resource includes pairs whose partners lack documented extracellular localization (e.g., calmodulin–channel and spectrin–phosphatase interactions). All per-pair biological interpretation in this paper uses extracellular-verified pairs (tiers 1 and 2, ∼90% of pairs; §4.28). Under this default filter, 14 of 16 originally significant combinations remained significant; the two that became marginal were both bipolar disorder (brain *P* = 0.056, hippocampus *P* = 0.057), consistent with the mixed synaptic and intracellular nature of adhesion signaling. A stricter secreted-ligand → membrane-receptor filter (tier 1 only; 54% pair retention) retained 8 of 16, with robust retention in heart and liver. In brain, the per-pair results after filtering converge on trans-synaptic adhesion (NRXN–NLGN) and neural adhesion–RTK signaling (NCAM1–FGFR1, L1CAM–CD9), which constitute the biological interpretation presented in §2.8. Fourth, Visium’s ∼55*µ*m resolution means each spot contains 1–10 cells; the “edges” therefore reflect communication between local cell populations rather than individual cell–cell contacts. Cross-resolution validation with cell-segmented Visium HD data (median nearest-neighbour distance ∼13*µ*m; §2.5) confirmed the liver LDL edge signal, and sender–receiver analysis at 8*µ*m resolution showed that all five plotted top liver–LDL pairs involve distinct cell types (e.g., hepatocyte-secreted F9 signalling to stellate-cell LRP1), consistent with intercellular rather than autocrine communication; single-cell platforms (MERFISH, Slide-seq) may further sharpen the decomposition. Fifth, the aggregate edge test for Alzheimer’s disease was non-significant in DLPFC, likely reflecting limited common-variant signal (mean *χ*^2^ = 1.26); per-pair analysis nonetheless recovered APP–LRP1 as a concentrated channel (§2.8). In heart, AD was aggregate-significant, with the top pairs (F8–LRP1, APP–LRP1) converging on LRP1-mediated clearance, consistent with the established role of lipoprotein trafficking in AD genetic architecture[50]. Integration with rare-variant data and microglia-enriched spatial datasets may reveal neuron–glia communication channels. Finally, EdgeMap quantifies heritability enrichment but does not identify causal variants; integration with fine-mapping and spatial causal mediation is a natural next step.

Looking ahead, the node–edge decomposition presented here illustrates a broader principle: genetic architecture has relational structure that is not captured by scoring cells in isolation, even when relevant tissues and cell types are correctly identified. Knowing that a tissue is enriched for heritability is the starting point; knowing which intercellular channels carry that heritability is the information needed to prioritize mechanistic follow-up. Concretely, the RTN4–CNTNAP1 paranodal axis nominated here for bipolar disorder predicts that genetic perturbation of this junction would alter cortical network synchrony—a hypothesis testable in conditional knockout models. As single-cell spatial technologies mature, extending the framework to additional tissue types, developmental time points, and perturbation contexts may reveal how communication heritability reshapes across disease states—connecting the “between-cell” biology of GWAS loci to targeted experimental design.

## 4 Methods

### 4.1 Spatial transcriptomics preprocessing

Spatial transcriptomics data were loaded from h5ad format and preprocessed using Scanpy[51]: genes expressed in fewer than 10 cells were removed, counts were library-size normalized to 10,000 per cell, and log1p-transformed. Spatial coordinates were extracted from the obsm[’spatial’] field.

### 4.2 Spatial graph construction

We constructed a *k*-nearest-neighbor graph (k=6) from spatial coordinates using Euclidean distance, where $p_{i}\in {\mathbb{R}}^{2}$ denotes the spatial coordinates of cell i . Edge weights follow a Gaussian kernel:

(1) $$w_{ij}=\exp\left(-\frac{\|p_{i}-p_{j}{\|}^{2}}{2\sigma^{2}}\right),$$

where $\sigma =d_{\max}/3$ and $d_{\max}$ is the maximum Euclidean distance for retaining a neighbor in the KNN graph (default 3,000*µ*m for 10x Visium). Self-edges are excluded $\left(w_{ii}=0\right)$ so that the communication score reflects exclusively intercellular (inter-spot) signaling. The weight matrix is symmetrized: $W\leftarrow \left(W+W^{⊤}\right)/2$ .

### 4.3 Ligand–receptor database

We used the LIANA Consensus resource[7], which integrates curated LR pairs from CellChatDB[16], CellPhoneDB, ICELLNET[52], connectomeDB2020, and CellTalkDB[53], filtered by literature support and protein localization (4,624 interactions). An LR pair was retained if every subunit gene was expressed in at least 5% of spots.

### 4.4 Spatial-weighted communication

Following established cell–cell communication scoring[16], for each LR pair p with ligand subunits $l_{p}$ and receptor subunits $r_{p}$, we computed per-cell communication intensity:

(2) $${\mathrm{comm}}_{p}(j)=R_{\text{eff}}^{\left(p\right)}(j)\cdot \sum_{i}w_{ij}L_{\text{eff}}^{\left(p\right)}(i),$$

where $L_{\text{eff}}^{\left(p\right)}(i)=\min_{g\in l_{p}}\left(\exp\left(x_{ig}\right)-1\right)$ and $R_{\text{eff}}^{\left(p\right)}(j)=\min_{g\in r_{p}}\left(\exp\left(x_{jg}\right)-1\right)$ are effective concentrations in natural scale ($x_{ig}$ is the log1p-normalized expression of gene g in cell i). The min over subunits implements the standard bottleneck model for heteromeric complexes[17].

### 4.5 Gene specificity score (GSS)

For each gene g, expression values were ranked across all n cells (one ranking per gene; ties broken arbitrarily). The gene-level GSS is the ratio of local to global geometric mean rank, maximized over all cells (max rather than mean, to capture the location where gene g is most spatially concentrated):

(2) $$\mathrm{GSS}(g)=\underset{i}{\max}\exp\left(\frac{1}{\left|N(i)\right|}\sum_{j\in N(i)}\log R_{jg}-\frac{1}{n}\sum_{j=1}^{n}\log R_{jg}\right),$$

where n is the total number of cells, $R_{jg}$ is the rank of cell j among all n cells for gene g (ascending; rank 1 = lowest expression, including zeros), and N(i) is the spatial neighborhood of cell i including i itself (|N(i)|=k+1 for interior cells).

### 4.6 Communication specificity score (CSS)

For each LR pair p, the pair-level score is the 95th percentile of per-cell communication specificity: $\mathrm{PairScore}(p)=Q_{95}\left(\left\{{\mathrm{comm}}_{p}(j)/{\mathrm{co}\overset{-}{m}m}_{p}\right\}\right)$. The 95th percentile captures the spatial concentration of communication in active niches while remaining robust to outliers (robust across the range 85–99%). Pairs with PairScore(p)≤1 are set to zero. The gene-level CSS is the maximum pair score across all LR pairs in which gene g participates (max ensures that a gene’s CSS reflects its strongest communication channel):

(4) $$\text{CSS}(g)=\underset{p:g\in l_{p}\cup r_{p}}{\max}\text{PairScore}(p).$$

Max aggregation follows the consensus of cell–cell communication tools (CellChat, CellPhoneDB): it ensures that a hub gene appearing in many pairs (e.g. ITGB1 in 456 pairs) receives the score of its strongest channel rather than an inflated sum. As a sensitivity analysis we also computed CSS using mean-over-pairs aggregation; landscape-level results were highly concordant (Spearman ρ=0.996, concordance of significance 83/85), confirming that the gene-level aggregation rule is not a critical degree of freedom. Note that per-pair significance testing (§4.8) operates on individual pair scores and is therefore entirely independent of the gene-level aggregation choice.

### 4.7 EdgeMap joint regression

Gene-level scores were mapped to SNP-level annotation LD scores using the precomputed SNP–gene weight matrix $W\in {\mathbb{R}}^{M\times G}$ from gsMap[6], whose entry $W_{sg}=\sum_{j\in \text{cis}(g)}r_{sj}^{2}$ aggregates squared LD correlations over all SNPs j in the cis-window of gene g:

(5) $$\ell_{s}^{\left(\text{node}\right)}=\sum_{g}W_{sg}\mathrm{GSS}(g),\;\ell_{s}^{\left(\text{edge}\right)}=\sum_{g}W_{sg}\mathrm{CSS}(g).$$

The joint regression extends the stratified LD score regression (S-LDSC) framework[2] by introducing these tissue-specific node and edge annotations alongside baseline annotations; the regression machinery and block-jackknife inference are unchanged. The model is:

(6) $$E\left[\chi_{s}^{2}\right]=1+N\sum_{q}\tau_{q}\ell_{s}^{\left(q\right)}+N\tau_{\text{node}}\ell_{s}^{\left(\text{node}\right)}+N\tau_{\text{edge}}\ell_{s}^{\left(\text{edge}\right)},$$

where $\left\{\ell_{s}^{\left(q\right)}\right\}$ are baseline LD score annotations and τ coefficients are estimated by weighted least squares with block-jackknife standard errors over ∼200 non-overlapping LD blocks. We report one-sided P values for $\tau_{\text{edge}}>0$, as our primary hypothesis is directional: we test for heritability enrichment in the edge annotation rather than depletion.

### 4.8 Per-pair conditional testing

For each LR pair p with communication specificity > 1.0, we constructed a pair-specific edge annotation (participating genes receive the pair’s score, all others zero), mapped it to SNP-level LD scores via **W**, and tested in a three-annotation model (baseline + node + single pair).

Because per-pair annotations are sparse (∼1,000 nonzero SNPs out of ∼1,000,000), the block-jackknife standard errors that underlie S-LDSC *z*-scores are poorly calibrated: most of the ∼200 LD blocks contain zero annotated SNPs and contribute no information, causing the null *z*-distribution to depart sharply from *N*(0,1) (empirical sd ≈ 4.4, excess kurtosis 50–5,000, KS *P* < 10^−100^; Extended Data Table 3), with a spike-and-slab structure reflecting the sparse annotation geometry. This is a known limitation of S-LDSC for small annotations[3].

We therefore calibrate per-pair significance via 50,000-replicate parametric null simulation per tissue. Under $H_{0}:\tau_{\text{node}}=0$, $\tau_{\text{pair}}=0$ (a global null in which only baseline LD structure generates association signal), we simulated GWAS $\chi^{2}$ statistics from the real baseline LD scores, ran the full per-pair S-LDSC pipeline for every testable pair, and recorded all null *z*-scores. Crucially, each pair accumulates its own 50,000-element null distribution, shaped by that pair’s specific annotation sparsity and LD structure; the null is therefore pair-specific, not pooled across pairs. The empirical *P*-value for each pair is

$$P_{\text{emp}}=\frac{n_{\text{null}\geq z_{\text{obs}}}+1}{N_{\text{null}}+1},$$

Because each pair’s null *z*-distribution is shaped by its sparse annotation pattern and is generally not centered at zero, this right-tail test identifies pairs whose observed *z* is shifted toward enrichment relative to the pair-specific null, regardless of the sign of the raw *z*-score. Because positive node enrichment could in principle alter the pair-level null through heteroskedasticity, we performed conditional-null sensitivity analyses across all eleven trait–tissue settings, simulating under each setting’s observed ${\hat{\tau }}_{\text{node}}$ (spanning a 130-fold range) with $\tau_{\text{pair}}=0$ (5,000 replicates each). Per-pair null standard deviations were nearly identical (median SD ratio 0.97–1.02, overall mean 1.00), and empirical *P*-value ranks were near-perfectly concordant (Spearman *ρ* ≥ 0.998), confirming that the global null provides adequate calibration (Extended Data Fig. ED4d,e). Bonferroni and Benjamini–Hochberg FDR correction were applied to the empirical *P*-values across all testable pairs within each tissue–trait combination. Pairs with null standard deviation < 0.5 were excluded as untestable (annotation too sparse). This assumption-free approach directly measures false-positive rates from the actual null distribution, regardless of its shape, yielding a minimum empirical *P* of 2.0×10^−5^ (50,001 replicates). Note that raw per-pair *z*-scores can be very large (e.g., *z >* 30) when a pair’s sparse annotation (∼2–10 nonzero SNPs) coincides with a major GWAS locus; such values reflect the amplifying effect of sparse annotations rather than proportionally large heritability contributions. The empirical *P*-value, which compares the observed *z* against the same pair’s null distribution under identical sparsity, is the appropriate significance measure.

#### Null $\chi^{2}$ generation.

Under $H_{0}$ each null $\chi^{2}$ statistic is generated as

(7) $$\chi_{s}^{2}=\left(1+N\sum_{q}{\hat{\tau }}_{q}\ell_{s}^{\left(q\right)}\right)Z_{s}^{2},\;Z_{s}\sim N(0,1),$$

where $\left\{{\hat{\tau }}_{q}\right\}$ are baseline coefficients estimated by S-LDSC from the observed GWAS summary statistics and $\left\{\ell_{s}^{\left(q\right)}\right\}$ are the baseline annotation LD scores. This preserves the full LD correlation structure across SNPs: the annotation-weighted LD scores encode pairwise LD between all SNPs in each block, so the synthetic $\chi^{2}$ values inherit the same dependence pattern as the real data. The trait-specific heritability enters through ${\hat{\tau }}_{q}$, which is estimated from the data rather than fixed at any pre-specified value; the fixed $h^{2}=0.3$ mentioned in the simulation study (§2.2) applies exclusively to the power analysis (Fig. 2), not to real-data inference. As a robustness check, we replaced the Gaussian noise with *t*-distributed noise ($Z_{s}\sim t_{5}$ ; excess kurtosis 6) and confirmed that per-pair null calibration is insensitive to heavy tails across all five tissues (median null-SD ratio 0.92; 90% of pairs within [0.5,1.5]; Extended Data Fig. ED7d).

### 4.9 Pattern-level significance

Because aggregate S-LDSC annotations have limited per-SNP variance, individual trait–tissue *z*-scores are inherently moderate; the primary statistical question is therefore whether the *pattern* of significant results across the landscape exceeds chance, rather than whether each combination independently survives global multiple-testing correction. We performed three pattern-level tests. (1) A one-sided binomial test comparing the number of significant combinations (k=16 of n=85 at α=0.05) against the null rate of 5%. (2) A trait-label permutation test for biological coherence: we defined 14 *a priori* expected positive pairings based on established tissue–trait biology, enumerated before examining results: heart × {SBP, DBP, CAD}; brain DLPFC × {SCZ, bipolar, BMI}; hippocampus × {BMI, bipolar}; liver × {LDL, TG, CAD, SBP, DBP}; gut × UC. Trait labels were shuffled across the 85 combinations 10^4^ times, and two statistics were compared to their null distributions: the count of significant results falling in expected pairings, and the mean edge *z*-score difference between expected and other pairings. (3) A Mann–Whitney U test comparing edge *z*-scores in expected pairings versus all others.

### 4.10 Edge gene definition

Throughout the downstream analyses below, *edge genes* are defined as all genes participating in at least one LR pair reaching FDR < 0.10 under empirical null calibration (§4.8); genes appearing in multiple pairs are counted once per trait–tissue combination. Biological-interpretation analyses (drug target enrichment, Mendelian enrichment) restrict edge genes to extracellular-verified pairs (tiers 1 and 2; §4.28), yielding 64 unique genes after pooling across significant combinations. Statistical analyses that assess GWAS signal strength (competitive gene-set test, gene-level method comparison) use all 72 edge genes regardless of tier, because these tests evaluate association enrichment rather than intercellular biology. All enrichment tests use the 1,877-gene LIANA Consensus LR database as the background set.

### 4.11 Drug target overlap analysis

Approved drug targets were obtained from DGIdb v4[46] via the GraphQL API, retaining genes with at least one interaction classified as having an approved drug (3,439 genes). Enrichment was assessed by one-sided Fisher’s exact test comparing the fraction of drug targets among edge genes to the background rate (826/1,877 = 44.0%). A pooled analysis combined unique edge genes across all significant combinations; a sensitivity analysis using all 72 edge genes (including tier 3) is reported in Supplementary Table 6.

### 4.12 Mendelian gene enrichment

Known causal genes for Mendelian forms of 15 complex traits were curated from OMIM. For each significant trait–tissue combination, we tested whether tier 1+2 edge genes are enriched for the corresponding Mendelian genes using a one-sided Fisher’s exact test. A pooled analysis combined unique edge genes across all significant combinations.

### 4.13 Locus-to-Gene enrichment

Locus-to-Gene (L2G) predictions were obtained from the Open Targets Platform (release 25.03)[22]. For each trait, we identified L2G-prioritized genes (score ≥ 0.5) from GWAS studies matching the trait by EFO disease identifiers and keyword search. Enrichment of edge genes among L2G-prioritized genes was tested by one-sided Fisher’s exact test against the LR database background, both per trait–tissue combination and pooled.

### 4.14 Competitive gene-set enrichment

To test whether edge genes carry stronger GWAS associations than expected from the LR database background, we performed a competitive gene-set test analogous to MAGMA[21]. For each trait, SNP-level *P*-values were mapped to genes using a ±10kb window, retaining the minimum *P*-value per gene. Gene-level *P*-values were −log_10_transformed to obtain association scores. For each significant trait–tissue combination, we compared the association scores of edge genes (all tiers) against all other genes in the LIANA Consensus LR database using a one-sided Wilcoxon rank-sum test.

### 4.15 Gene-level method comparison

To quantify the complementarity of EdgeMap with existing gene prioritization methods, we compared edge genes (all tiers) against two standard approaches for each of the 16 significant trait–tissue combinations. (1) *MAGMA-like gene-level testing:* for each trait, SNP-level *P*-values were mapped to genes within a ±10kb window using the same procedure as the competitive gene-set test above; genes reaching Bonferroni significance (*P* < 2.5 × 10^−6^, correcting for ∼20,000 protein-coding genes) were classified as MAGMA-significant. Gene coordinates were obtained from Ensembl GRCh37 (release 87) and SNP positions from the 1000 Genomes EUR phase 3 reference panel. (2) *Open Targets L2G:* for each trait, L2G-prioritized genes (score ≥ 0.5) were obtained using per-trait EFO and keyword matching as described above, restricted to studies of the *same* trait (i.e., per-trait matching rather than pooled across all traits as in the enrichment analysis). An edge gene was classified as “found by MAGMA or L2G” if it appeared in either method’s gene list for the specific trait in which it was identified as an edge gene. The unique fraction was computed as the proportion of edge genes not found by either method.

### 4.16 Simulation study

We generated synthetic GWAS $\chi^{2}$ under controlled architectures using real annotation LD scores from spatial transcriptomics. Expected $\chi^{2}:E\left[\chi_{s}^{2}\right]=1+N\left(\sum_{q}\tau_{q}\ell_{s}^{\left(q\right)}+\right.\left.\tau_{\text{node}}\ell_{s}^{\left(\text{node}\right)}+\tau_{\text{edge}}\ell_{s}^{\left(\text{edge}\right)}\right)$, where baseline coefficients $\left\{\tau_{q}\right\}$ were held fixed at values estimated from the data (assuming $h^{2}=0.3$ distributed uniformly across baseline annotations) and $\chi_{s}^{2}$ were sampled as $E\left[\chi_{s}^{2}\right]\cdot Z_{s}^{2}$ with $Z_{s}\sim N(0,1)$ . Four scenarios (null, node-only, edge-only, mixed) × 500 replicates, with $\tau =5\times 10^{-9}$ for active annotations and N=340,159 . Simulations were conducted independently on heart, brain DLPFC, and gut annotation LD scores to verify that calibration is not tissue-specific (Extended Data Table 3).

### 4.17 Data sources

Human heart spatial transcriptomics data were obtained from the 10x Genomics Visium platform (4,247 spots; discovery section) and from Kuppe et al. 2022[24] (4 control Visium sections: P1, P7, P8, P17; replication). Human brain DLPFC Visium data were obtained from Maynard et al. 2021[18] (discovery: section 151507, 4,226 spots; replication: 11 additional sections 151508–151676 from 3 donors). Hippocampus Visium data were obtained from Thompson et al. 2025[19] (GSE264692, section V10B01–085 A1, 4,992 spots). Liver Visium data were obtained from Guilliams et al. 2022[20] (GSE192741; discovery: section JBO001; replication: JBO002–JBO004; gene names were converted to human orthologs by uppercase mapping). Cross-resolution validation used cell-segmented Visium HD liver data from Yakubovsky & Itzkovitz 2025[27] (Zenodo 10.5281/zenodo.17735506; samples M2 and M6 from healthy live donors; 50,278 and 25,671 cells, respectively). Intestine Visium data were obtained from Gupta et al. 2024[54] (GEO: GSE189184; discovery: sample B4, 2,575 spots; replication: samples B8, B10, C5).

GWAS summary statistics were grouped as follows. Cardiovascular traits: systolic and diastolic blood pressure from UK Biobank (N=340,159), atrial fibrillation from Nielsen et al.[55] $\left(N_{\text{eff}}=228,221\right)$, heart rate from UK Biobank (*N* = 340,162), and coronary artery disease from van der Harst & Verweij[56] ($N_{\text{eff}}=380,832$ ; independent replication: FinnGen Release 10[26], $N_{\text{eff}}=166,436$, no UK Biobank overlap). Psychiatric and neurological traits: schizophrenia from PGC3[57] $\left(N_{\text{eff}}=233,471\right)$, major depression from Howard et al.[58] (*N* = 807,553), bipolar disorder from PGC3[59] $\left(N_{\text{eff}}=101,962\right)$, ADHD from PGC[60], and Alzheimer’s disease from Bellenguez et al.[50] (N=788,989) . Metabolic and immune traits: BMI from Yengo et al.[61] (N=681,275), ulcerative colitis and Crohn’s disease from de Lange et al.[62] (UC: N=45,975 ; CD: N=40,266), LDL cholesterol and triglycerides from UK Biobank (independent replication: LDL from the Global Lipids Genetics Consortium[25], N=173,082, no UK Biobank overlap), T2D from FinnGen r10[26], and height from UK Biobank.

Baseline LD scores (baselineLD v2.2), HapMap3 SNPs, and the SNP–gene weight matrix were obtained from the gsMap resource repository[6, 63].

### 4.18 Cross-dataset replication analysis

To assess reproducibility, we ran the full EdgeMap pipeline independently on 12 brain DLPFC sections (151507–151676, from 3 donors[18]), 5 heart Visium sections (1 from 10x Genomics plus 4 control sections P1, P7, P8, P17 from Kuppe et al.[24]; 5 donors), 4 liver Visium sections (JBO001–JBO004, from 4 donors[20]), and 4 gut Visium sections (B4, B8, B10, C5, from 4 donors). Each section was processed independently through spatial graph construction, LR communication scoring, annotation LD score computation, and S-LDSC regression. Replication was quantified at three levels: (1) aggregate-level: fraction of sections reaching *P* < 0.05 (one-sided) per trait–tissue pair; (2) random-effects meta-analysis using the DerSimonian–Laird estimator[64], with edge *z*-scores as effect sizes (SE = 1); under the null, the S-LDSC coefficient *z*-score is asymptotically *N*(0,1)[2], making this parameterization equivalent to Stouffer’s method when $\tau^{2}=0$ ; (3) annotation stability: Spearman correlations of per-pair communication intensity score vectors across all section pairs, quantifying whether the same LR pairs dominate the communication landscape regardless of section. For independent-GWAS replication, the same tissue-derived annotation LD scores were paired with substitute GWAS summary statistics (GLGC 2013 LDL[25] for liver; FinnGen Release 10 CAD[26] for heart) and the full EdgeMap pipeline was rerun; per-pair concordance was assessed via Spearman correlation of *z*-score vectors across the common LR pairs present in both runs.

### 4.19 Visium HD processing

Cell-segmented Visium HD liver data[27] (samples M2 and M6) were preprocessed using the same Scanpy pipeline as standard Visium (library-size normalization to 10,000, log1p transformation, 5% expression threshold). The spatial neighbor graph was constructed with k=6 nearest neighbors, identical to the standard Visium pipeline. For aggregate edge testing, each cell-segmented bin was treated as one spatial unit. Cell-type labels for the sender–receiver analysis (§4.20) were assigned by maximum expression of canonical marker genes: ALB (hepatocyte), ACTA2 (stellate cell), CD68 (Kupffer cell), KRT19 (cholangiocyte), and PECAM1 (endothelial cell).

### 4.20 Sender–receiver cell-type assignment

For the top-ranked liver–LDL edge pairs, we tested whether ligands and receptors localize to distinct cell types using the Visium HD M6 section at native 8*µ*m bin resolution (475,519 bins). Bins were assigned to cell types (hepatocyte, stellate, Kupffer, cholangiocyte, endothelial) based on maximum marker gene expression. For each pair, the “primary sender” was the cell type with the highest mean ligand expression (excluding cell types with zero expression), and the “primary receiver” was the cell type with the highest mean receptor expression. Pairs were classified as intercellular if the primary sender and receiver cell types differed.

### 4.21 Spatial gradient analysis

For each tissue with significant edge heritability, we tested whether communication intensity of top-ranked LR pairs varies non-randomly along the tissue’s primary spatial axis. In brain, the spatial axis was defined by cortical layer annotations (layer guess from Maynard et al.). In liver and gut, the axis was defined as the first principal component of spatial coordinates, approximating the portal–central and crypt–villus axes, respectively. In heart, PC1 of spatial coordinates was used.

For each top-ranked LR pair (FDR < 0.10 under empirical null calibration), we computed the Spearman correlation between per-spot communication intensity and the spatial axis coordinate. The aggregate test compared the observed mean absolute correlation across these pairs to a null distribution obtained by permuting spatial axis labels 10^3^ times. The enrichment ratio is the observed mean divided by the permutation mean.

### 4.22 Edge module decomposition

To identify groups of LR pairs with coordinated trait-loading profiles, we constructed a pair × trait *z*-score matrix from per-pair conditional testing across all trait–tissue combinations with significant aggregate edge heritability. Pairs present in fewer than two significant combinations were excluded. We applied hierarchical clustering (Ward’s linkage, Euclidean distance) to the standardized *z*-score matrix and selected the number of modules k that maximized the silhouette score over k∈{5,6,7,8} (optimal k=5, silhouette = 0.38). Module–trait loadings were computed as the mean per-pair *z*-score within each module for each trait.

### 4.23 Edge relevance correlation (ERC)

For each pair of traits with significant edge heritability in at least one shared tissue, we computed the Spearman correlation of their per-pair *z*-score vectors within that tissue. When multiple tissues were available, we averaged the within-tissue correlations. The resulting ERC matrix was compared to published genome-wide genetic correlations $\left(r_{g}\right)$ from LD score regression by computing the Spearman rank correlation across all 17 trait pairs for which both ERC and $r_{g}$ values were available.

### 4.24 Node–edge orthogonality analysis

To quantify the independence of node and edge heritability signals, we computed per-gene node specificity scores (GSS) and edge communication scores (CSS) for each tissue using the discovery section. For each significant trait–tissue combination, we extracted the set of genes participating in FDR < 0.10 LR pairs under empirical null calibration (“edge genes”) and the top-100 genes ranked by node specificity (“node genes”). We quantified overlap via the Jaccard index and assessed whether edge genes were ranked differently in the node distribution using the rank-biserial correlation from the Mann–Whitney U test. Genome-wide concordance between GSS and CSS was measured by Spearman correlation across all genes.

### 4.25 Cell-type composition baseline control

To test whether edge heritability is confounded by cell-type composition, we augmented the S-LDSC regression with spatial domain annotations. For each tissue, spots were grouped into K spatial domains: in brain DLPFC (K=8), we used the published cortical layer assignments[18]; for other tissues (K=10), domains were identified by Leiden clustering on a spatial nearest-neighbour graph (six nearest neighbours, resolution 0.8). For each domain d, we computed a gene-level specificity score ${\text{specificity}}_{dg}=\left({\bar{x}}_{dg}-{\bar{x}}_{g}\right)/s_{g}$, where ${\bar{x}}_{dg}$ is the mean expression of gene g in domain d and ${\bar{x}}_{g}$, $s_{g}$ are the genome-wide mean and standard deviation. These domain-specificity vectors were mapped to SNP-level LD scores via the same SNP–gene weight matrix **W** used for node and edge annotations, yielding K additional annotation columns $\ell_{\text{domain},d}$ . We then ran S-LDSC with baseline + node + edge + domain annotations and compared edge *z*-scores with and without domain control. Separately, conditioning on gene-level cis-LD burden (a proxy for gene length and local LD architecture) and mean expression—the two most commonly cited technical confounders in LD-score-based enrichment—retained 13 of 14 significant settings (mean *z*-score change −5.6%), consistent with the domain-conditioning results above.

### 4.26 Ligand–receptor specificity test

To test whether the edge annotation captures signal specific to known ligand–receptor biology rather than generic spatial co-expression, we constructed null annotations by replacing each gene in the LIANA Consensus database with a non-LR gene matched on expression level and spatial coefficient of variation. For each tissue, we computed per-gene mean expression ${\bar{x}}_{g}$ and spatial CV $cv_{g}=\text{sd}\left(x_{g}\right)/\left({\bar{x}}_{g}+\epsilon \right)$, then partitioned genes into 20 × 20 joint-quantile bins on these two axes. Each LR gene was replaced by a random non-LR gene drawn from the same bin, preserving expression magnitude and spatial variability while removing LR identity. Each shuffled database was processed through the full EdgeMap pipeline (spatial communication, CSS, annotation LD scores, S-LDSC) with the same GWAS and spatial graph as the real analysis. We performed 500 shuffles for each of four trait–tissue combinations (heart × SBP, brain × bipolar disorder, liver × LDL, gut × ulcerative colitis) and computed empirical *P*-values as the fraction of shuffled aggregate edge *z*-scores exceeding the real value.

### 4.27 Locus ablation test

To test whether edge heritability is driven by a small number of strong GWAS loci, we performed greedy locus clumping: SNPs were sorted by $\chi^{2}$, the top SNP was designated as a lead, all SNPs within ±500kb on the same chromosome were assigned to that locus and removed from the pool, and the procedure was repeated to identify K independent loci. For each ablation level (K∈{1,5,10,20}), we excluded locus SNPs from the GWAS summary statistics and re-ran the S-LDSC regression with the same annotation LD scores. SNP genomic positions were obtained from the HapMap3 LD weight reference files.

### 4.28 Subcellular localization quality control

Ligand–receptor databases curated for cell–cell communication inference occasionally include protein–protein interactions whose partners lack documented extracellular localization (e.g., calmodulin–ion-channel and spectrin–phosphatase complexes that operate intracellularly). Such pairs can pass the per-pair statistical test when their constituent genes coincide with GWAS loci, but do not represent intercellular signaling. To distinguish intercellular communication channels from intracellular co-regulation, we classified all LIANA Consensus genes by UniProt subcellular location annotations (865 secreted, 928 membrane, 60 intracellular, 24 unknown) and assigned each LR pair to one of three tiers:

1. **Paracrine (secreted** → **membrane):** ligand classified as secreted, receptor as membrane-anchored. These pairs represent the highest-confidence intercellular interactions.
2. **Contact-dependent (membrane** → **membrane):** both partners membrane-anchored (e.g., neurexin–neuroligin, ephrin–Eph). These require direct cell–cell contact.
3. **Localization-unverified:** at least one partner is classified as intracellular or unknown (e.g., CALM–CACNA1C, SPTAN1–PTPRA). These pairs are reported for completeness but excluded from biological interpretation.

Tiers 1 and 2 are collectively termed *extracellular-verified* because both partners are localized to the cell surface or secretory pathway; biological interpretation of perpair results (§2.6–§2.9) is restricted to these tiers. As robustness checks, we reran the full EdgeMap pipeline under two filtering stringencies for all 16 originally significant trait–tissue combinations: (i) tiers 1 and 2 jointly (∼90% pair retention), retaining 14 of 16 significant results; and (ii) tier 1 only (54% pair retention), retaining 8 of 16.

### 4.29 Software and reproducibility

The EdgeMap pipeline is implemented in Python using NumPy, SciPy, Scanpy[51], and scikit-learn. The pipeline processes one trait × one tissue in approximately 23 seconds including per-pair testing.

## Extended Data

**Extended Data Fig. ED3:**
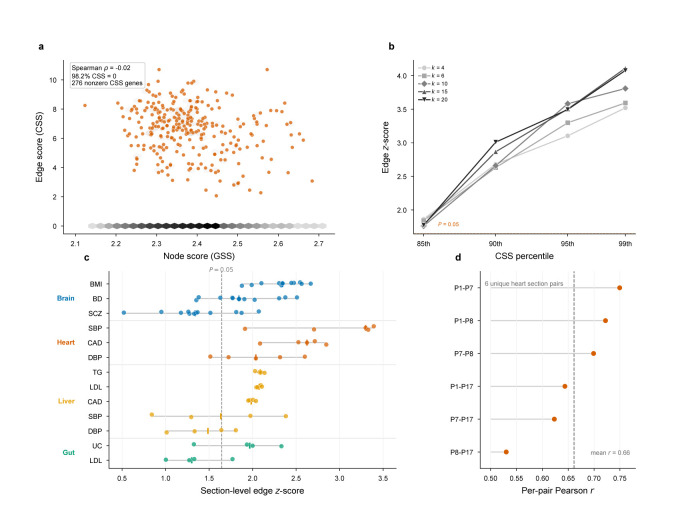
Technical diagnostics: orthogonality, parameter sensitivity, and annotation-level stability. **a**, Gene specificity score (GSS, node) vs. communication specificity score (CSS, edge) for all 15,217 genes in heart; Spearman *ρ* = −0.02, confirming near-complete orthogonality. **b**, Parameter sensitivity: edge *z*-scores for heart × SBP across spatial neighborhood sizes (k∈{4,6,10,15,20}) and communication specificity percentiles (85th–99th). All 20 combinations are significant (*P* < 0.05, one-sided), with *z*-scores increasing monotonically with the percentile threshold. **c**, Cross-section replication: section-level edge *z*-scores across independent tissue sections (12 DLPFC, 5 heart, 4 liver, 4 gut) for all tested trait–tissue combinations. Points denote sections, horizontal lines denote the observed range, short vertical ticks denote the median, and the dashed line marks *z* = 1.645. **d**, Annotation-input stability: per-pair communication-intensity Pearson correlations for the 6 unique heart section pairs (mean *r* = 0.66), confirming that LR communication scores are reproducible before entering S-LDSC.

**Extended Data Fig. ED4:**
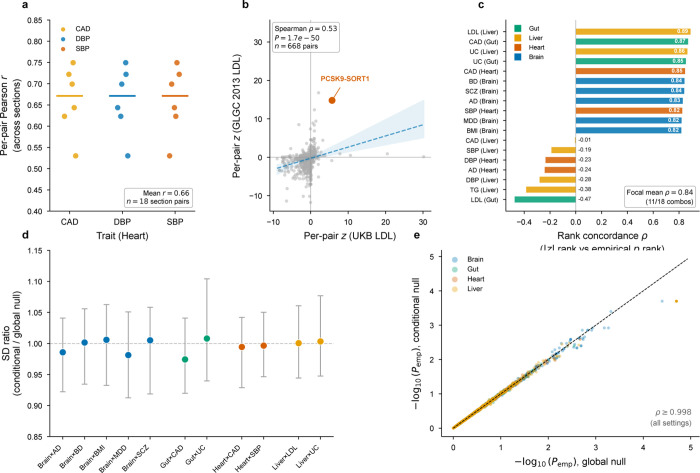
Pair-level robustness: stability across sections, cohorts, calibration methods, and null conditioning. **a**, Per-pair *z*-score Pearson correlations across independent heart tissue sections (4 Kuppe et al. donors × 3 traits; mean *r* = 0.66). **b**, Per-pair *z*-score scatter for liver × LDL between UK Biobank and GLGC 2013 GWAS (no sample overlap; 668 common pairs; Spearman *ρ* = 0.53, *P* = 1.7 × 10^−50^). **c**, Rank concordance between raw |z| and empirical *P*-value across all tested pairs for each tissue–trait combination (Spearman *ρ*; mean = 0.84). **d**, Median per-pair null standard-deviation ratio (conditional/global) across all eleven trait–tissue settings (point: median; bars: interquartile range). All medians fall within 2% of unity, indicating that conditioning on the observed node enrichment has negligible effect on pair-level null width. **e**, Empirical *P*-value concordance under the global $\left(\tau_{\text{node}}=0\right)$ and conditional $\text{(}\tau_{\text{node}}={\hat{\tau }}_{\text{node}}\text{)}$ nulls across all settings (Spearman *ρ* ≥ 0.998).

**Extended Data Fig. ED5:**
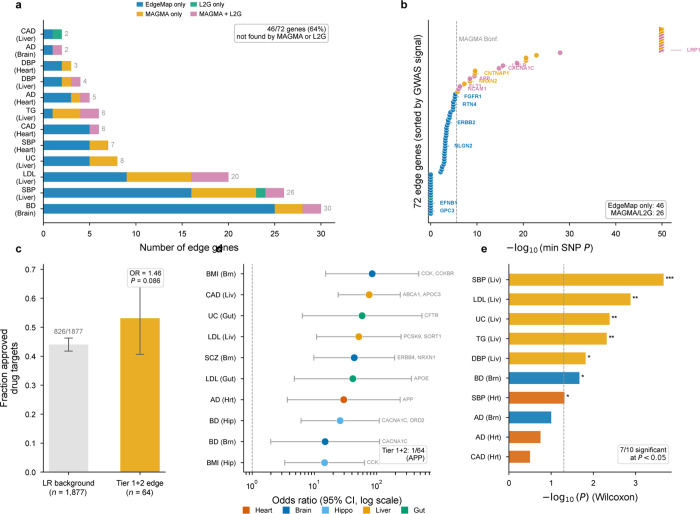
Biological characterization and uniqueness of edge genes. **a**, Stacked bar chart showing, for each tissue–trait combination with FDR < 0.10 edge genes, the number of edge genes found by MAGMA (orange), L2G (green), both (purple), or EdgeMap only (blue); 46 of 72 edge genes (64%) were not identified by either method. **b**, Each edge gene plotted by its strongest nearby GWAS signal (−log_10_ of minimum SNP *P* within ±10kb), coloured by discovery method. **c**, Drug target overlap: fraction of tier 1+2 edge genes that are approved drug targets versus LR background (OR = 1.46, *P* = 0.086). **d**, Mendelian gene overlap across trait–tissue combinations, shown as odds ratios on a log scale. **e**, Competitive gene-set test (−log_10_ *P*, Wilcoxon); edge genes carry stronger GWAS associations than LR background in 7 of 10 combinations.

**Extended Data Fig. ED6:**
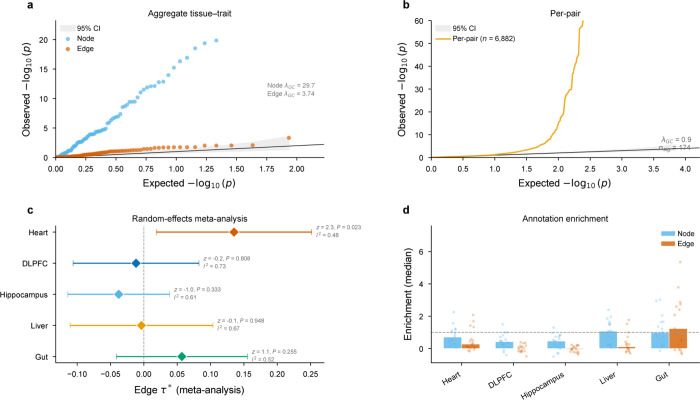
Aggregate enrichment quantification and null calibration. **a**, QQ plot of aggregate tissue–trait *P*-values (*n* = 85 combinations). Both node $\left(\lambda_{\text{GC}}=29.7\right)$ and edge $\left(\lambda_{\text{GC}}=3.74\right)$ annotations show clear enrichment above the null diagonal, with a 95% beta-distribution confidence envelope (grey). **b**, QQ plot of per-pair empirical *P*-values (*n* = 6,882 pairs). The bulk of tests is well-calibrated $\left(\lambda_{\text{GC}}=0.9\right)$, consistent with the empirical-null procedure; a pronounced upper tail yields 174 Bonferroni-significant pairs. **c**, Random-effects meta-analysis of standardised effect size $\tau^{*}$ across 17 traits per tissue (DerSimonian–Laird). Node $\tau^{*}$ is significant in all five tissues (z=4.0−7.4) ; edge $\tau^{*}$ reaches significance only in heart $\left(\tau_{\text{meta}}^{*}=0.14,z=2.3,P=0.023\right)$, reflecting the tissue–trait specificity of intercellular communication signals. **d**, Median node and edge enrichment (fold-change over genomic background) per tissue, with individual tissue–trait combinations overlaid.

**Extended Data Fig. ED7:**
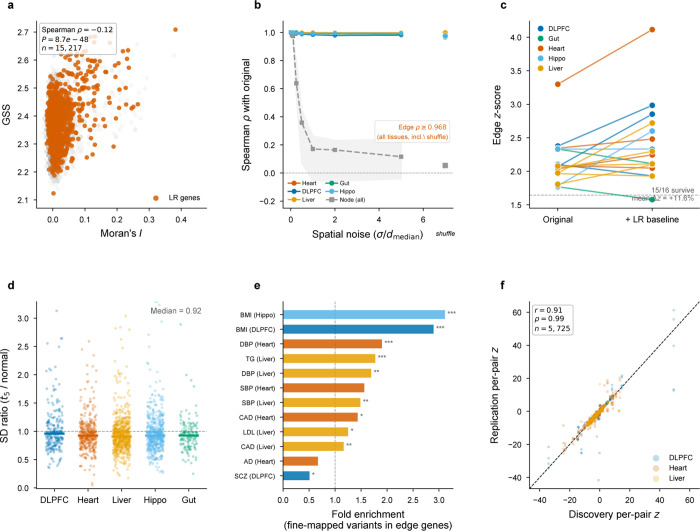
Extended robustness and validation analyses. **a**, Moran’s I (spatial autocorrelation) vs. gene specificity score (GSS, spatial concentration) for 15,217 genes in heart (Spearman *ρ* = −0.12), confirming that GSS captures a distinct spatial property. LR genes (orange) are highlighted. b, Coordinate noise robustness across all five tissues: Spearman *ρ* between original and noise-perturbed scores as a function of noise level $\left(\sigma /d_{\text{median}}\right)$ . Edge scores remain stable (ρ≥0.97) across all noise levels including complete coordinate shuffle, while node scores collapse (ρ→0), ruling out node-to-edge leakage. c, Effect of adding an LR membership baseline annotation to S-LDSC: paired slope plot of edge *z*-scores before (left) and after (right) conditioning on binary LR membership. 15 of 16 significant combinations survive (mean *z*-score change: +11.6%). d, Heavy-tailed null robustness: SD ratio of per-pair null distributions generated with $t_{5}$ vs. Gaussian effect-size distributions, by tissue (median = 0.92; 90% within [0.5,1.5]). e, Fine-mapped variant enrichment: fold-enrichment of SuSiE credible-set PIP in edge-annotated vs. background genes. Significant in 9 of 12 testable combinations (Mann–Whitney *P* < 0.05), providing validation orthogonal to S-LDSC. f, Cross-section per-pair *z*-score scatter: discovery vs. replication sections pooled across available tissue–trait combinations, confirming reproducibility of pair-level signals.

## Supplementary Material

Supplement 1

Supplement 2

Supplement 3

Supplement 4

## Data Availability

GWAS summary statistics are publicly available: UK Biobank Neale Lab round 2 (http://www.nealelab.is/uk-biobank; SBP, DBP, BMI, height, heart rate, T2D); ICBP[65] (SBP, DBP); CARDIoGRAMplusC4D[66] (CAD); GLGC[25] (LDL, TG); PGC3[67] (SCZ); PGC (bipolar, major depression); IGAP[68] (AD); FinnGen release 6 (UC, Crohn’s disease). Spatial transcriptomics datasets are available from GEO and ArrayExpress: heart (Kuppe et al.[24], GSE165838); brain DLPFC (Maynard et al.[18], http://research.libd.org/spatialLIBD/); hippocampus (Thompson et al.[19], GSE264692); liver (Guilliams et al.[20], GSE192742); gut (Gupta et al.[54], SCP1884); Visium HD liver (10x Genomics demonstration datasets). The LIANA Consensus ligand–receptor database (v0.1.13) is available at https://github.com/saezlab/liana. Baseline LD scores and SNP–gene weight matrices are available from the gsMap resource repository[6].

## Code availability

The EdgeMap pipeline is available at https://github.com/cafferychen777/EdgeMap under an open-source license. Scripts to reproduce all analyses and figures are included in the repository.
